# Quality of life and self-perceived health of adolescents in Middle School

**DOI:** 10.1590/2317-1782/20212021046

**Published:** 2022-03-18

**Authors:** Graziela Nunes Alfenas Fernandes, Stela Maris Aguiar Lemos

**Affiliations:** 1 Departamento de Fonoaudiologia, Faculdade de Medicina, Universidade Federal de Minas Gerais – UFMG - Belo Horizonte (MG), Brasil.

**Keywords:** Adolescent, Quality of Life, Self Concept, Health, Socioeconomic Factors, Adolescente, Qualidade de Vida, Autoimagem, Saúde, Fatores Socioeconômicos

## Abstract

**Purpose:**

Quality of life and self-perception of health are useful indicators for children and adolescents, as they are associated with objective and subjective states of health, including physical, cognitive, social, emotional, and environmental aspects. This study aims to characterize the adolescents at a Brazilian private financing school and to analyze the association between quality of life, health self-perception, and the sociodemographic profile.

**Methods:**

This is a cross-sectional, observational study conducted with 124 elementary school students. The questionnaires of Characterization of the participants, Health Self-perception and Pediatric Quality of Life Inventory ™ (PedsQL™) were applied to the adolescents and, to the parents or guardians, the Brazil Economic Classification Criteria questionnaires and PedsQLTM.

**Results:**

The majority of the participants were female, belonged to economy class A, declared to have excellent health self-perception, and attributed a good score for their health. The majority of parents and adolescents evaluated positively the physical, psychosocial, and overall quality of life dimensions. Having a positive health self-perception increased the chances of having a good quality of life and the increase of one year in age decreased the chances of the adolescent having a positive quality of life assessment.

**Conclusion:**

The study of adolescence is essential to broaden the understanding of aspects related to the quality of life, both in its physical and psychosocial dimension and to self-perception. This knowledge will provide adolescents with an incentive for their well-being, better performance of their activities, and greater preparation for adult life.

## RESUMO

**Objetivo:**

A qualidade de vida e a autopercepção de saúde são indicadores úteis associados a estados objetivos e subjetivos de saúde, incluindo aspectos físicos, cognitivos, sociais, emocionais e ambientais. Este estudo caracterizou adolescentes de uma escola brasileira de financiamento privado e analisou a associação entre qualidade de vida, autopercepção de saúde e perfil sociodemográfico.

**Método:**

Trata-se de estudo observacional, analítico e transversal, realizado com 124 adolescentes estudantes do ensino fundamental. Foram aplicados os questionários de Caracterização dos participantes, Autopercepção de saúde e Pediatric Quality of Life Inventory™ (PedsQL™) aos adolescentes e, aos pais ou responsáveis, os questionários Critério de Classificação Econômica Brasil e PedsQLTM.

**Resultados:**

A maioria dos participantes era do sexo feminino, pertencia à classe econômica A, declararam ter autopercepção de saúde excelente e atribuiu boa nota para sua saúde. A maior parte dos pais e dos adolescentes avaliou positivamente as dimensões física, psicossocial e qualidade de vida geral. Ter uma autopercepção positiva da saúde aumentou as chances de ter uma qualidade de vida boa e o aumento de um ano na idade diminuiu as chances de o adolescente ter uma avaliação positiva da qualidade de vida.

**Conclusão:**

O estudo da adolescência e suas peculiaridades é fundamental para ampliar a compreensão de aspectos relacionados à qualidade de vida, tanto em sua dimensão física quanto psicossocial e à autopercepção de saúde. Esse conhecimento permitirá proporcionar aos adolescentes o incentivo para o seu bem-estar, melhor desempenho de suas atividades e maior preparo para a vida adulta.

## INTRODUCTION

Adolescence is a phase of large physical and psychosocial changes. Quality of life (QoL) is an indicator that seeks to advance in the understanding of the multiple dimensions of health status as perceived by healthy individuals, of all age groups and cultures^(1,2)^. Such an indicator is especially useful in children and adolescents taking into account the importance of having a good self-perception for a better performance of the daily functions.

The term quality of life, in its generic sense, is best known through the concept proposed by the World Health Organization (WHO), and the individuals’ perception of their position in life, in the context of the culture and value systems in which they live, and about their goals, expectations, standards, and concerns^(3)^. Urzúa^(4)^ defines the health-related quality of life (HRQoL) as the level of wellbeing derived from the individual assessment of the various domains of their life, considering the impact they have on their health status. The concepts of health and disease have a close relationship with the cultural context of an era; as well as the social, political, and economic aspects of a people. The history of the evolution of these concepts is interesting and shows the transformation of ideas in this area of ​​human experience^(5)^. Thus, health and disease are complex and multifactorial processes, also related to personal experiences and lifestyles. By its subjectivity and multidimensionality, the state of health, when evaluated by the individual guarantees greater validity and reliability^(6)^.

Research articles reveal important and concordant findings regarding QoL and health self-perception in adolescence^(7,8)^. They indicate that the low score in the perception of QoL in adolescents is possibly related to the evolutionary stage in which the adolescents are, characterized by an increased pace of life, new responsibilities and ways of doing things, impatience about their independence, and warnings from their elders^(9)^. A European study using the Kidscreen instrument that addresses 10 dimensions (health and physical activity, feelings, general mood, self-perception, leisure time, family and family environment, economic issues, friends, school environment, and learning and bullying) compared boys with girls concerning perceptions of health-related quality of life and found differences in most dimensions. Boys presented usually higher average scores compared to girls, except for the dimension “school environment and learning”, in which girls present higher average values. In the case of the “economic” and “friends” dimensions, gender differences are not significant^(10)^. In Brazilian study with adolescents from public and privately funded schools in the state of Rio de Janeiro, highlighted the influence of external factors such as socioeconomic, family, school, and social factors on HRQOL, where older adolescents, public school students and those with less possession of goods had lower HRQL values in most dimensions^(11)^. In another study, it was observed that the fact that when adolescents do not feel able to perform physical activities and do not have the time to socialize with their friends, these are factors that contribute to a low perception of health-related quality of life^(1)^.

Taking into account the needs of the adolescence phase, strategies should be sought to making known the potential of this population, collaborating for communication between professionals and action planning in health care based on comprehensiveness, multidisciplinary approach, and intersectionality, associated with a policy aimed towards health promotion and protection, development of health education practices, identification of risk groups, early detection of injuries, treatment and rehabilitation^(12-14)^.

As the quality of life, the health self-perception, and the sociodemographic profile of the adolescent students of high school are associated. The particularities of this association are questions that guide this investigation. The knowledge about the interaction of these factors can contribute to a better understanding of aspects, not only related to health but also referred to behavior and school performance that are inherent to this stage of life. This will allow planning actions that aim at improving the quality of life in all its dimensions. The objective of this study was to investigate the association between quality of life, health self-perception, and socio-demographic profiles of adolescents enrolled in middle school.

## METHODS

### Participants

The Ethics Committee, through opinion 2,422,795, approved the project of this study. Parents/guardians and adolescents who agreed to participate signed the Informed Consent Form and the Informed Approval Form, respectively.

This is an observational, cross-sectional study encompassing a sample stratified by gender, age, and school year composed of 124 Brazilian adolescents aged 11 to 14 enrolled in middle school of a private financing school.

As inclusion criteria were considered: adolescents enrolled in the institution researched in Middle School, aged between 11 and 14 years. It was used as an exclusion criterion for the non-fulfillment of the research instruments.

The sample calculation was based on a 15% estimate of poor quality of life in the population using PedsQL™^(15)^, an index fund in the literature used as a reference for the calculation of sample size^(16)^. A final sample of 114 individuals was estimated to obtain 80% statistical power considering a 9% sampling error and 95% confidence interval. The precision used in the sample calculation was 15% and the significance level was 5%. The analysis was done using the Test for One Proportion in the Minitab 14 Release software.

### Instrumentation and procedures

The instruments used were the Brazilian Economic Classification Criteria-CCEB^(17)^, Pediatric Quality of Life Inventory - PedsQL™ 4.0. and the Health Self-perception Questionnaire. The first, with 15 items, was answered by the parents/guardians of the adolescents surveyed, aimed to group the participants into classes ranging from A (higher purchasing power) to E (lower purchasing power) according to the possession of material goods and the level of education of the head of the family.

The second instrument, PedsQL™ 4.0, composed of 23 questions, answered by parents/guardians and adolescents, assessed QoL in four domains: physical (physical dimension), emotional, social, and school (psychosocial dimension).

The third instrument, prepared by the researchers and answered by the adolescents, verified the health self-perception, through three questions: “How do you evaluate/ consider your health these days? “What note would you give to your health?” The answers to the first question were rated using a Likert scale with the following options: very bad, bad, regular, good, and excellent. For the answer to the second, the numerical scale was used with values from 0 to 10, considering 0 very poor and 10 excellent.

Data collection was performed between June and August 2018 through forms created in Google Forms and applied in the adolescents' time and school environment.

### Data analysis

The adolescents' characterization information, such as gender, age, school year, economic classification, and self-perception of health as explanatory variables, and quality of life assessed in four dimensions (physical, emotional, social, and school) were considered as the response variables.

For the data analyses, the information of the answered forms was inserted into a database and a series of descriptive analyses of the variables were carried out employing absolute and relative frequency distribution of the categorical variables while a numerical synthesis was performed regarding the continuous ones. The following tests were used: Pearson's chi-square test and Fisher's exact test for categorical variables to evaluate the association between response variables and explanatory variables. The variables that had associations with a significance level of 20% (p≤0.20) with the response variables were considered to enter into the logistic regression models. Due to the high correlation of the variables, it was chosen to use multivariate logistic regression models. Six initial models were constructed, namely: Physical Dimension (parents), age and health self-perception; Psychosocial dimension (parents) and health self-perception (univariate, since only the age variable was associated with this dimension); General quality of life (parents), age and health self-perception; Physical Dimension (adolescents), gender and health self-perception; Psychosocial Dimension (adolescents), age and health self-perception; and general quality of life (adolescents), gender, age, and health self-perception.

For the evaluation of the associations in the final models, the significance level of 5% and 95% confidence intervals were considered. The Statistical Package for the Social Sciences (SPSS), version 21.0 was used for all the analysis processes.

## RESULTS

The majority of adolescents were female, showing the highest frequency in the 11-year-olds (27.4%), although all age groups were near, ranging from 22.6% to 27.4% of the total. As for schooling, 32.3% of the adolescents were in the 6th grade and 66.9% were in the economic class A. 55.6% of the adolescents stated that they had excellent health self-perception and 44.4% attributed a grade 9 to their health. The median health score was 9, presenting an average of 8.85 and SD = 1.183.

The parents' evaluation showed that the dimensions with the highest averages were physical (80.42, SD = 19.32) and social (83.99, SD = 15.33), while the lowest were the emotional and school dimensions, both with a mean of 67.50 (SD = 12.92 and SD = 16.21, respectively). In the adolescents' evaluation, the highest average occurred in the social dimension, 85.97; SD = 12.60, and the lowest average, 62.02; SD = 16.81 was in the emotional dimension (Table 1).

**Table 1:** Descriptive Analysis of PedsQL^TM^ Variables (N=124)

**Dimensions**	Median	Average	Standard Deviation
**Parents assessment**			
Physical	87.50	80.42	19.32
Emotional	65.00	67.50	12.92
Social	90.00	83.99	15.33
School	65.00	67.50	16.21
Psychosocial	76.67	75.73	11.28
General Quality of Life	79.35	77.36	12.61
**Adolescents’ assessment**			
Physical	78.25	77.23	7.79
Emotional	60.00	62.02	16.81
Social	90.00	85.97	12.60
School	75.00	75.52	14.69
Psychosocial	75.00	74.50	11.07
General Quality of Life	78.26	78.35	9.64

Source: Own elaboration based on collected data

To facilitate the analysis of bivariate association we chose to re-categorize the explanatory variables: CCEB; health self-perception and score attributed to health. Thus, CCEB showed a distribution of 67% of adolescents in class A and 33% in classes B1 and B2. The health self-perception was good or excellent in 91% of the adolescents while 74% evaluated the health score above 8 (Table 2). PedsQL^TM^ variables were also distributed in two categories, low and high, based on the values of the medians of the physical, psychosocial, and overall quality of life scores, according to the parents’ and adolescents' responses, and the frequency distributions of these variables are presented separately: 52% of the adolescents evaluated positively the physical, psychosocial and overall quality of life dimensions. In the parents' evaluation, the physical dimension 56% showed positive evaluation, the psychosocial dimension, 53%; and finally, in the quality of life there was a 51% positive evaluation (Table 3).

**Table 2:** Descriptive Analysis of CCEB, Health Self-perception and Health Scores in Two Categories (N=124)

**CCEB**		
	**N**	**%**
A	83	67
B1/B2	41	33
Total	124	100
**Health self-perception**		
	**N**	**%**
Bad/Fair	11	9
Good/Excellent	113	91
Total	124	100
**Health score**		
	**N**	**%**
≤8	32	26
>8	92	74
Total	124	100

Caption: CCEB = Brazilian Economic Classification Criteria

Source: Own elaboration based on collected data

**Table 3:** PedsQL^TM^ Variables in Categories (N=124)

**Physical dimension - adolescents**		
	**N**	**%**
≤78.25	64	52
>78.25	60	48
Total	124	100
**Psychosocial dimension - adolescents**		
	**N**	**%**
≤75.0	65	52
>75.0	59	48
Total	124	100
**General Quality of Life - adolescents**		
	**N**	**%**
≤78.26	65	52
>78.26	59	48
Total	124	100
**Physical dimension - parents**		
	**N**	**%**
≤87.5	69	56
>87.5	55	44
Total	124	100
**Psychosocial dimension - parents**		
	**N**	**%**
≤76.67	66	53
>76.67	58	47
Total	124	100
**General Quality of Life – parents**		
	**N**	**%**
≤79.35	63	51
>79.35	61	49
Total	124	100

Source: Own elaboration based on collected data

Regarding the parents' responses, at the 20% level of significance, the following variables were associated with the physical dimension: age, school year, health self-perception and health score; to the psychosocial dimension, the variable health self-perception; and to the general quality of life, the variables age, school year, health self-perception and health score (Table 4).

**Table 4:** Bivariate Analysis of Association Between PedsQL^TM^ Variables (Parents and Adolescents) and Selected Variables (N=124)

**Characteristics**	**Parents**						**Adolescents**					
	**Dimensions**						**Dimensions**					
	Physical		Psychosocial		General Quality of Life		Physical		Psychosocial		General Quality of Life	
	low	high	low	high	low	high	low	high	low	high	low	high
	**N (%)**	**N (%)**	**N (%)**	**N (%)**	**N (%)**	**N (%)**	**N (%)**	**N (%)**	**N (%)**	**N (%)**	**N (%)**	**N (%)**
**Gender**												
Female	38 (55.1)	29 (52.7)	36 (54.5)	31 (53.4)	32 (50.8)	35 (57.4)	40 (62.5)	27 (45.0)	33 (50.8)	34 (57.6)	32 (49.2)	35 (59.3)
Male	31 (44.9)	26 (47.3)	30 (45.5)	27 (46.6)	31 (49.2)	26 (42.6)	24 (37.5)	33 (55.0)	32 (49.2)	25 (42.4)	33 (50.8)	24 (40.7)
p-value	0.795		0.903		0.462		0.051		0.444		0.260	
**Age**												
11	15 (21.7)	19 (34.5)	17 (25.8)	17 (29.3)	14 (22.2)	20 (32.8)	17 (26.6)	17 (28.3)	14 (21.5)	20 (33.9)	14 (21.5)	20 (33.9)
12	16 (23.2)	14 (25.5)	16 (24.2)	14 (21.4)	15 (23.8)	15 (24.6)	15 (23.4)	15 (25.0)	14 (21.5)	16 (27.1)	15 (23.1)	15 (25.4)
13	19 (27.5)	13 (23.6)	15 (22.7)	17 (29.3)	18 (28.6)	14 (23.0)	15 (23.4)	17 (28.3)	18 (27.7)	14 (23.7)	17 (26.2)	15 (25.4)
14	19 (27.5)	9 (16.4)	18 (27.3)	10 (17.2)	16 (25.4)	12 (19.7)	17 (26.6)	11 (18.3)	19 (29.2)	9 (15.3)	19 (29.2)	9 (15.3)
p-value*	0.054		0.398		0.170		0.508		0.028		0.042	
**School Year**												
6º	17 (24.6)	23 (41.8)	18 (27.3)	22 (37.9)	14 (22.2)	26 (42.6)	20 (31.2)	20 (33.3)	17 (26.2)	23 (39.0)	17 (26.2)	23 (39.0)
7º	18 (26.1)	16 (29.1)	19 (28.8)	15 (25.9)	18 (28.6)	16 (26.2)	14 (21.9)	20 (33.3)	15 (23.1)	19 (32.2)	15 (23.1)	19 (32.2)
8º	18 (26.1)	8 (14.5)	15 (22.7)	11 (19.0)	19 (30.2)	7 (11.5)	16 (25.0)	10 (16.7)	16 (24.6)	10 (16.9)	16 (24.6)	10 (16.9)
9º	16 (23.2)	8 (14.5)	14 (21.2)	10 (17.2)	12 (19.0)	12 (19.7)	14 (21.9)	10 (16.7)	17 (26.2)	7 (11.9)	17 (26.2)	7 (11.9)
p-value*	0.022		0.265		0.059		0.298		0.014		0.014	
**CCEB**												
A1	48 (69.6)	35 (63.6)	44 (66.7)	39 (67.2)	42 (66.7)	41 (67.2)	42 (65.6)	41 (68.3)	46 (70.8)	37 (62.7)	46 (70.8)	37 (62.7)
B1/B2	21 (31.4)	20 (36.4)	22 (33.3)	19 (32.8)	21 (33.3)	20 (32.8)	22 (34.4)	19 (31.7)	19 (29.2)	22 (37.3)	19 (29.2)	22 (37.3)
p-value	0.486		0.946		0.948		0.749		0.341		0.341	
**Health self-perception**												
Bad/fair	9 (13)	2 (3.6)	8 (12.1)	3 (5.2)	9 (14.3)	2 (3.3)	9 (14.1)	2 (3.3)	9 (13.8)	2 (3.4)	9 (13.8)	2 (3.4)
Good/excellent	60 (87)	53 (96.4)	58 (87.9)	113 (91.1)	54 (85.7)	59 (96.7)	55 (85.9)	58 (96.7)	56 (86.2)	57 (96.6)	56 (86.2)	57 (96.6)
p-value	0.11**		0.174		0.031		0.036		0.041		0.041	
**Health score**												
≤8	22 (31.9)	10 (18.2)	20 (30.3)	12 (20.7)	21 (33.3)	11 (18.0)	22 (34.4)	10 (16.7)	26 (40.0)	6 (10.2)	26 (40.0)	6 (10.2)
>8	47 (68.1)	45 (81.8)	46 (69.7)	92 (74.2)	42 (66.7)	50 (82.0)	42 (65.6)	50 (83.3)	39 (60.0)	53 (89.8)	39 (60.0)	53 (89.8)
p-value	0.083		0.222		0.052		0.024		<0.001		<0.001	

* Linear-by-linear association;

** Fisher Exact Test

Caption: CCEB = Brazilian Economic Classification Criteria

Regarding the results of the PedsQL^TM^ parents’ version, no significant association was found for models 1 and 2. In model 3, the variable health self-perception was associated with the general quality of life. This finding suggests that perceiving health as good or excellent increased by 4.92 times the chance of having a good quality of life.

Analyzing the PedsQL^TM^ responses according to the adolescents' perception, in the final model 4, no significant association was found. In the final model 5, age was inversely associated with the psychosocial dimension. A one-year increase in age decreased the adolescents’ chances of having a positive psychosocial dimension by 31%. In the final model 6, age was associated with the general quality of life. A one-year increase in age decreased the adolescents’ odds of having a positive quality of life assessment by 28%. The suitability of the models was evaluated using the Hosmer and Lemeshow test. All the final models presented a good fit (Table 5).

**Table 5:** Results of Multiple Logistic Regression Analysis Between PedsQL^TM^ Variables (Parents and Adolescents) and Selected Variables. Initial and Final Models (N=124)

	Dimensions PedsQLTM	Models	Characteristics		
			Female gender	Age (years)	Health self-perception Good/Excellent
Parents	Physical	Initial Model 1	−	0.74 (0.53-1.03)	3.67 (0.75-17.96)
		Final Model 1	−	0.73 (0.53-1.01)	−
	Psychosocial	Final Model 2	−	−	2.53 (0.63-10.02)
	General Quality of Life	Initial Model 3	−	0.82 (0.59-1.13)	4.66 (0.96-22.66)
		Final Model 3	−	−	**4.92 (1.02-23.77)** *
Adolescents	Physical	Initial Model 4	1.99 (0.96-4.14)	−	4.58 (0.94-22.47)
		Final Model 4	2.04 (0.99-4.17)	−	−
	Psychosocial	Initial Model 5	−	0.71 (0.51-0.98)*	4.22 (0.86-20.66)
		Final Model 5	−	0.69 (0.50-0.96)*	−
	General Quality of Life	Initial Model 6	0.63 (0.30-1.32)	0.73 (0.53-1.01)	4.49 (0.91-21.15)
		Final Model 6	−	0.72 (0.52-0.98)*	−

Reference Categories: Gender = Female; School Year = 9th grade; Health self-perception = bad/fair

* P<0.05

Initial/final Model fitness (Hosmer and Lemeshow): Model 1, final p=0.989; Model 2 −; Model 3 final p=0.992; Model 4, final p=0.708; final; Model 5, final p=0.933; Model 6, final p=0.849

Caption: PedsQL™ = Pediatric Quality of Life Inventory

Source: Own elaboration based on collected data

Considering the PedsQL^TM^ result according to the parents' perceptions, the variable health self-perception was associated with the general quality of life. Regarding the PedsQL^TM^ result according to the adolescents' perceptions, age was associated with the psychosocial dimension and general quality of life.

## DISCUSSION

Economic and sociocultural factors may interfere with health-related quality of life^(6,18)^. The objective of this study was to understand the association between quality of life, health self-perception, and socioeconomic conditions, considering such information useful for formulating strategies for disease prevention and health promotion. Studies show that estimating a prevalence of approximately 15% of children with low quality of life, two-thirds of these children come from financially underprivileged families^(16)^. The participants of this study are middle school students from a private school in the Center-South region of Belo Horizonte and this explains the fact that more than two-thirds of the participants belong to A-class according to the CCEB. Sampling stratification and study population size provided a representative sample from the point of view of gender, age, and school year for the researched setting.

Health self-perception is associated with the objective or subjective state of health, including physical, cognitive, and emotional aspects^(19)^. This study showed that 91% of adolescents evaluated their health as good or excellent. Despite the lack of statistical significance for the gender variable, the data showed the strong tendency of female participants to assess health worse. A school-based epidemiological survey conducted in 2011 with a sample of 6,261 adolescents aged 14 to 19 years from publicly funded schools in 48 municipalities in the Brazilian Northeast evidenced a worse health perception among female participants^(20)^. In a 2012 study that investigated the topic, 3,920 people among adolescents, adults, and seniors in the southern region of Brazil obtained results that indicate that women their health in lower grades than men in all life cycles^(21)^. A study carried out with Portuguese adolescents revealed that boys have a better perception of quality of life, as do younger adolescents and those who live with their parents (mother and father)^(22)^. Thus, it is possible to indicate that the results of the present study corroborate the literature regarding the stronger negative perception of health in women. This can be explained by the fact that they are more attentive about health care, have a greater sensitivity to perceive physiological changes, and understand health more greatly, encompassing physical, mental, and social aspects^(23,24)^.

Lifestyle is an important health indicator and personal and environmental aspects of self-perception should also be taken into account^(25)^. Encouraging the development of adolescents' social skills through the establishment of public policies can become a factor in the youth's engagement in self-care^(26)^. Thinking about the educational environment, school health programs can promote physical and mental health to improve the quality of life of students^(27)^. The quality of life measured by the PedsQL^TM^ version for parents and adolescents evaluates four dimensions: physical, emotional, social, and school and for analysis of association, the physical and psychosocial dimensions (emotional, social, and school) were considered.

In this study, perceiving health as good or excellent increased the chance of by 4.92 times the chance of having a good quality of life. Regarding the parents' point of view on the quality of life of adolescents, the higher evaluations were found in the physical and social dimensions, while the emotional and school dimensions had worse evaluations. The adolescents assessed their quality of life rating the social dimension as best while the emotional dimension had the worst evaluation. The validation study of the Brazilian version of the PedsQL^TM^ 4.0 instrument, conducted in a hospital in São Paulo included 120 healthy children and adolescents aged 8 to 18 years and their parents. The research indicated excellent reliability in the internal consistency of the tool as well as identifying significant correlations between the perspectives of the children and parents, especially in the physical dimension^(28)^.

It is possible to consider that parents and adolescents perceive more easily the aspects contained in the questions of the physical and social dimensions and therefore, possibly better evaluated. Emotional factors, including feelings, have a less concrete nature, and because adolescence is a transitory phase, fraught with doubts and anxieties, suggesting a worse evaluation. Schooling is importante in studies on the quality of life of adolescentes. The analysis showed the worst results in the schooling domain as per the parents’ perspective, corroborating earlier studies that suggest the decreased interest in school subjects as they progress along the educational trajectory. There is evidence that younger students express a greater interest and motivation to fulfill their homework, compared with older ones, which reinforces the need to think of new and more motivating strategies to stimulate learning^(29)^. Regarding the age variable from the perspective of the adolescent, it was inversely associated with the psychosocial dimension as well as it was associated with the general quality of life. A one-year increase in age decreased by 28% of the adolescents’ odds to have a positive quality of life assessment. A one-year increase in age decreased the adolescents’ chances to have a positive psychosocial dimension by 31%. The literature indicates that with increasing age, adolescents begin to understand health as something more complex, multifactorial, not restricted to the absence of diseases. The challenges and responsibilities brought by the arrival of adulthood are also a cause of anxiety and insecurity, interfering with the quality of life and health self-perception^(24)^.

Due to the characteristics of the sample and since adolescents belong mostly to the economic A-class, the results of this investigation cannot be generalized, and subsequent comparison with adolescents of different sociodemographic conditions may be convenient to broaden the conclusions. The limitations of this study may be related to the cross-sectional design used, because it is not possible to establish a causal relationship between the dimensions of PedsQL^TM^ and the other aspects surveyed. Furthermore, the instrument does not allow for a wide description and analysis of contextual aspects of adolescents^(30)^.

## CONCLUSION

The research showed the close relationship between the health self-perception and the quality of life of adolescent students, reinforcing the evidence that emerged from previous research. The elements emerging from the analysis performed by this study generate reflections for the advancement of knowledge in the areas of health and education.

Because of the above, it is interesting to investigate the age group of adolescence, its peculiarities, and the implications of such aspects on the quality of life, both in its physical and psychosocial dimension, that englobes emotional, social, and school aspects, of these individuals and in the health self-perception. It is evident the crucial need to provide adolescents with incentives for their well-being, as a means to better performance of their activities, including a better relationship with themselves and others.
